# Editorial: Novel horizons in pediatric food allergy

**DOI:** 10.3389/fped.2022.1036371

**Published:** 2022-10-13

**Authors:** Simona Barni, Betul Buyuktiryaki

**Affiliations:** ^1^Allergy Unit, Department of Pediatrics, Meyer Children's Hospital, Florence, Italy; ^2^Division of Pediatric Allergy, Koc University School of Medicine, Istanbul, Turkey

**Keywords:** alpha-gal syndrome, eosinophilic esophagitis, food allergy, human milk, oral immunotherapy, pediatric

## Introduction

Food allergy is a global health concern affecting millions of people worldwide. In recent years, interest in this field has provided novel findings on the prevention, diagnosis, and management of allergies, thus, opening new horizons in our knowledge of this subject.

## Definition and classification of food allergy

A food allergy (FA) is an abnormal hypersensitivity immune response to a given food allergen that occurs reproducibly (1). The immune response can be classified based on the immune system components involved in non-IgE-mediated and IgE-mediated FA (1). The clinical manifestations of IgE-mediated FA and non-IgE mediated FA occur, respectively, within or later than 2 h from food ingestion (2).

An exception to this classification is represented by the alpha-gal syndrome (AGS) described by Saretta et al. Indeed, the AGS has unusual characteristics: it is a delayed hypersensitivity reaction due to the ingestion of red meat (from non-primate mammals) but clinically immediate. Moreover, the production of specific IgE is directed against a disaccharide sugar called galactose-α-1,3-galactose (α-gal) expressed on the surface of mammalian cells, and not against protein as is usually the case in food allergies. Lastly, the AGS does not appear after every ingestion and could occur for food, until then, tolerated. In most cases, a positive clinical history of tick bites is present months before the onset of symptoms. AGS should be kept in mind in every patient with a history of idiopathic urticaria or anaphylaxis and unclear allergic reactions with unusual timing or characteristics.

## Prevention of FA

In recent years preventive measures for reducing the risk of FA have been focused on research (3), in particular the effect of breastfeeding on the development of FA in the offspring (4). The mini-review edited by Kosmeri et al. summarizes the latest evidence regarding allergen characteristics in human milk that may induce oral tolerance: the allergen amount in the breastmilk depends on the mother's diet, the antigen shedding in the human milk depends on the antigen, and it may follow a different kinetic between women. Moreover, the allergenicity of the food allergen in human milk may be influenced by the mode of food consumption, fresh or cooked. Although the dose of food allergen in human milk is very tiny, in the range of nanograms per milliliter, a tolerogenic effect has been found. Furthermore, the immune status of the mother may play a role in oral tolerance induction since the presence of antigen-specific immunoglobulins and immune complexes provide positive effects in allergy prevention. Although much progress has been made in the field of prevention, there is some interesting news on breastfeeding and food allergy prevention that needs to be better understood in order to develop new strategies for preventing the development of food allergy in the offspring.

## Management

Currently, there is no cure for FA other than a strict elimination diet of the culprit food (5). This type of attitude, defined as “passive,” is not exempt from the risk of a possible allergic reaction, even severe, due to the accidental intake of the trigger food (5). Hence, an “active approach” to increase the allergen reactivity dose to prevent life-threatening reactions is urgently needed such as oral immunotherapy (OIT) as described by Akarsu et al. The authors provide a critical overview of the current evidence on OIT in children with FA. The main goal of OIT is “sustained unresponsiveness” or tolerance meaning it is possible to safely assume any dose of the incriminate food, even after a long period of its avoidance. If the tolerance is not reached, we talk about desensitization, which is the ability to intake the culprit food safely due to a regular daily intake of the same food. While previous studies revealed a reduction in sIgE levels, an increase in IgG4 levels, suppression of basophils and mast cells, and the role of Treg cells, the mechanism of OIT is not completely understood. The OIT is mainly conducted for cow's milk, hen's egg, and peanut in children that are 4–5 years old, although lately a few reports have described OIT with other allergens, such as tree nut, wheat, and sesame.

There is an intense debate on the utility of OIT in children: on the one hand, the ability to increase the reactivity threshold dose and the achievement of a desensitization state as a possible outcome, and on the other, the possibility of an adverse reaction during the build-up and maintenance phase, even if mild to moderate, as well as the distant prospect of reaching tolerance.

Omalizumab, an anti-IgE monoclonal antibody, has been used as an adjuvant during OIT protocol to reduce the risk of an allergic reaction, as described by Akarsu et al. In milk, egg, and peanut OIT, omalizumab has been shown to reduce the number and the severity of reactions by reaching the build-up phase faster. Despite this, the efficacy of omalizumab in OIT treatment remains to be clarified, especially the effectiveness after its discontinuation.

Additionally, a rare but possible long-term side effect of OIT is eosinophilic esophagitis (EoE). Eosinophilic esophagitis (EoE) is a chronic allergic inflammatory disease of the esophagus, manifesting with the symptoms of esophageal dysfunction. The review by Votto et al. focuses on diet therapy for the management of EoE, considering first-line treatment using international guidelines. The diet therapy comprises an elemental diet (ED) or food elimination diet (FED). The ED consists in removing all foods and feeding the patients with an amino acid-based formula for at least 6 weeks. It is the most effective treatment with a significant reduction of symptoms and an achievement of histological remission in 90% of pediatric cases. The ED is the cornerstone treatment for severe EoE. In children with high diet restrictions due to multiple food allergies, the ED can be used as a nutritionally complete diet for adequate growth. Despite its advantages, the ED also has limits: high cost, poor palatability, and low patient compliance, and the intake through nasal gastric or gastric tube feeding may induce feeding skill regression.

The FED consists of the elimination of one or more groups of food, in particular milk, wheat, egg, soy/legumes, peanut/tree nuts, and seafood/fish. In general, the more foods are eliminated from the diet, the more likely it is that remission is achieved. Foods should be avoided both in fresh and baked forms.

Diet therapy is a successful treatment but is limited by low patient compliance, the need for several endoscopies, food restriction with potential nutritional deficiency, and negative impact on the patient's quality of life. For these reasons, patients with EoE must be managed by a multidisciplinary team.

Moreover, EoE has a clinical heterogeneity, as indicated by Votto et al. Thus, attempts have been made to determine the phenotypes and the endotypes of EoE based on the symptoms, endoscopic and histological findings, and response to treatments, which would be helpful for physicians to decide on the optimal treatment, either diet or medication, and provide a personalized approach for each patient.

## Conclusion

This Research Topic strove to provide an overview of recent progress in the field of food allergy research. Our four review articles provide novel findings in the field of clinical manifestation, along with the prevention and treatment of food allergies.

## Author contributions

SB and BB wrote and contributed substantial intellectual contributions to this Editorial. All authors contributed to the article and approved the submitted version.

## Conflict of interest

The authors declare that the research was conducted in the absence of any commercial or financial relationships that could be construed as a potential conflict of interest.

## Publisher's note

All claims expressed in this article are solely those of the authors and do not necessarily represent those of their affiliated organizations, or those of the publisher, the editors and the reviewers. Any product that may be evaluated in this article, or claim that may be made by its manufacturer, is not guaranteed or endorsed by the publisher.
